# Role of dexmedetomidine in modifying immune paralysis in patients with septic shock: randomized controlled trial

**DOI:** 10.1186/s40635-023-00542-2

**Published:** 2023-09-04

**Authors:** Mohamed Elayashy, Eman A. Elsayed, Ahmed M. Mukhtar, Sahar Kasem, Sara A. Elmetwally, Sara Habib, Walaa Abdelfattah, Doaa Ghaith, Amr Hussein

**Affiliations:** 1 Department of Anesthesia and Intensive Care, Kasr Al-Ainy Faculty of Medicine, Cairo University, Cairo, Egypt; 2 Department of Clinical Pathology, Kasr Al-Ainy Faculty of Medicine, Cairo University, Cairo, Egypt

**Keywords:** Dexmedetomidine, Septic shock, Sedation, Immune paralysis

## Abstract

**Background:**

Immune paralysis can be defined as a hypoinflammatory state associated with the incapacity of the immune system to release proinflammatory mediators despite the clearance of pathogens by antimicrobials. Persistent immune paralysis leads to failure to eradicate primary infections with a substantial increase in the risk of multiorgan dysfunction and mortality. The state of immune paralysis is caused mainly by the diminished ability of monocytes to release proinflammatory cytokines in response to endotoxin. This phenomenon is known as endotoxin tolerance. This study aimed to assess the role of dexmedetomidine in modifying immune paralysis in septic shock patients.

**Methods:**

Twenty-four patients with septic shock were randomized into two groups of 12 patients. A continuous intravenous infusion of dexmedetomidine started at 0.15 µg kg^−1^ hr^−1^ and adjusted by 0.15 µg kg^−1^ h^−1^ to a maximum of 0.75 µg kg^−1^ h^−1^ (10 ml h^−1^), while midazolam was started at 1 mg h^−1^ (2 mL hr^−1^) and adjusted by 1 mg h^−1^ to a maximum of 5 mg h^−1^ (10 mL h^−1^). All infusions were adjusted by increments of 2 mL/hr^−1^ to maintain blinding. Serum levels of CD42a+/CD14+, HLADR+/CD14+, CRP, IL-6, IL-10 and TNF-α were measured at baseline (T1), 12 h (T2), and 24 h (T3).

**Results:**

Treatment with dexmedetomidine yielded no significant difference in CD42a+/CD14+, HLADR+/CD14, CD24b-MFI, HLADR-MFI, IL6 and TREM1 at all time points when compared with midazolam treatment. There was no significant difference in TLR levels between the two groups. Cardiac output in the dexmedetomidine group showed a significant decrease at 6, 12 and 24 h (P = 0.033, 0.021, and 0.005, respectively) compared with that in the midazolam group.

**Conclusion:**

Our results indicated that dexmedetomidine did not affect CD42a+/CD14+ and HLA-DR+/CD14+ expression in septic patients. Furthermore, cytokine production and inflammatory biomarkers did not change with dexmedetomidine infusion.

*Trial registration* Clinical trial.gov registry (NCT03989609) on June 14, 2019, https://register.clinicaltrials.gov.

## Background

Immune paralysis is defined as a hypoinflammatory state associated with incapacity of the immune system to release pro-inflammatory mediators despite the clearance of pathogens by antimicrobials. Persistent immune paralysis leads to failure to eradicate the primary infection with a substantial increase in the risk of multiorgan dysfunction and mortality.

The state of immune paralysis is caused mainly by the reduced capacity of monocytes to respond to endotoxin by releasing proinflammatory cytokines. This phenomenon is known as endotoxin tolerance [1]^.^ The effect of endotoxin tolerance on leukocytes causes an increase in the release of immunosuppressive mediators, mainly interleukin-10 (IL-10), and a decrease in antigen presentation as a result of reduced expression of human leukocyte antigen-DR (HLA-DR). Furthermore, there is evolving evidence that platelets contribute to inflammation and the initial host defense response [2]. Following activation, platelet-derived microparticles (PMPs) are released from the surface of platelets and are responsible for the induction of inflammation. One of these microparticles is CD42.

Dexmedetomidine is a selective α2-adrenergic receptor agonist. It has sedative, anxiolytic, sympatholytic and hemodynamic stability characteristics [3]. α-2 adrenergic receptor agonists have effects on immunity, inflammation, and coagulation [4]. Dexmedetomidine treatment can successfully reduce the generation of inflammatory mediators and promote beneficial effects on endotoxemic animal microcirculation, according to experimental research on septic rats [3, 4]. Furthermore, Zhou et al. investigated the effect of dexmedetomidine on CD42/CD14, HLA-DR + /CD14 + and inflammatory cytokine levels in patients undergoing multilevel spinal fusion. They found that dexmedetomidine infusion was associated with a significant decrease in CD42/CD14, increased HLA-DR + /CD4 + and marked decreases in IL-6 and tumor necrosis factor-alpha (TNF-alpha) compared with the control group and concluded that DEX may alleviate immunosuppression in patients undergoing multilevel spinal fusion [5].

Therefore, the effects of dexmedetomidine on CD42a + /CD14 + and HLADR + /CD14 + levels, as well as the inflammatory cytokines TNF-α, IL-6 and IL-10, in septic shock patients were evaluated in the current study. We hypothesized that dexmedetomidine would have a suppressive effect on the inflammatory response and inflammatory mediators in septic shock patients.

## Methods

This prospective randomized controlled trial was conducted at Cairo University hospitals after receiving ethical approval from the Faculty of Medicine Research Ethics Committee (Committee Chairperson: Professor M. Mohsen Ibrahiem) on 18 May 2019 (N-5-2019). The study was registered on the clinical trial.gov registry (NCT03989609) on June 14, 2019. The study period was June 2019 to August 2021. After obtaining written informed consent, patients were randomly assigned into two groups: the dexmedetomidine group (Group I: n = 12) and the midazolam (control) group (Group II: n = 12). All patients above 18 years old who were mechanically ventilated, suspected to have had septic shock (cases in which the patient required vasopressor treatment after fluid resuscitation to keep the mean arterial pressure at 65 mm Hg) and a surgical source of sepsis was controlled by evacuation of infected fluid and/or removal of necrotic tissue. Patients with acute hepatitis, severe liver disease (Child‒Pugh class C), a left ventricular ejection fraction less than 30%, a heart rate less than 50 beats/min, 2nd or 3rd degree heart block, allergy to dexmedetomidine or midazolam and pregnancy were excluded from the study. Randomization was performed using block randomization and concealed using a sequentially numbered, sealed opaque envelope. The double-blinded study was ensured by having independent physicians not participating in the study to prepare the midazolam or dexmedetomidine in a ready-to-inject form by dilution in 50 ml 0.9% saline.

### Study protocol

All patients were monitored with noninvasive arterial blood pressure, five-lead electrocardiography (ECG), pulse oximetry, and invasive arterial pressure obtained from a radial arterial catheter.

Upon ICU admission, according to our institutional protocol, a fluid responsiveness test was performed for all enrolled patients to determine the need for fluid therapy (fluid responsiveness is defined as an increase in the SV by 15% after infusing 500 ml crystalloids). Fluid boluses were repeated until the patients became fluid unresponsive. If the mean arterial blood pressure (MAP) remained < 65 mmHg after administration of the initial fluid bolus, norepinephrine infusion was titrated to maintain the MAP at 65 mmHg.

#### Sedation protocol

All patients received analgesia with fentanyl at a fixed dose of 0.5 µg kg^−1^ hr^−1^. Each patient received the study drug within 6 h after ICU admission. Depth of sedation was assessed using the Richmond Agitation and Sedation Scale (RASS) score [6], which ranges from − 5 (unarousable) to + 4 (combative), without initiating a loading dose. Group I patients received dexmedetomidine (0.075 µg kg^−1^ mL^−1^), and group II patients received midazolam (0.5 mg mL^−1^). Both drugs were prepared in 0.9% saline in a 50 mL syringe indistinguishable from one another. Both agents were titrated to maintain the RASS range of − 3 to − 1. Dexmedetomidine infusion started at 0.15 µg. kg^−1^ hr^−1^ and adjusted by 0.15 µg kg^−1^ h^−1^ to a maximum of 0.75 µg kg^−1^ hr^−1^ (10 ml h^−1^), while midazolam started at 1 mg hr^−1^ (2 mL hr^−1^) and adjusted by 1 mg.hr^−1^ to a maximum of 5 mg h^−1^ (10 mL h^−1^). All infusions were adjusted by increments of 2 mL/hr^−1^ to maintain blinding. Patients in either group not adequately sedated by study drug titration received a bolus dose of fentanyl 0.5–1 µg kg. Assessment of the RASS score was performed every 2 h and prior to any dose of rescue therapy. The study drugs were infused for 24 h, and then, the choice of sedation was determined according to the preference of the attending physician.

### Data collection

Patient characteristics: age, sex, body mass index (BMI), cause of ICU admission, source of sepsis, APACHE II score 24 h after admission, and daily SOFA score for the first five days in the ICU were recorded.

#### Hemodynamic variables

Heart rate, mean arterial blood pressure, CVP, and cardiac output (CO) were monitored with a LIDCORapid (Masimo LIDCO ™ CA, USA). All hemodynamic parameters were measured continuously and recorded at baseline before drug administration and then at 6 h, 12 h and 24 h thereafter.

#### Cytokine levels

Serum samples were withdrawn, separated immediately and kept at − 20 °C for cytokine measurement. IL-6 and TREM-1 serum levels were measured by human premixed multianalyte technology (Human Magnetic Luminex Assay from R&D system, UK). All measurements were performed at baseline before drug administration and 12 and 24 h after drug administration.

#### Flow cytometry

Fresh EDTA anticoagulated blood samples were obtained for CD42, HLA-DR and TLR4 expression on monocytes. The expression of CD42a+/CD14+, HLA-DR+/CD14+ and TLR4+/CD14+ was detected by flow cytometry (FACSCanto cytometer from BD).

Other data collected:

- C reactive protein (CRP).
- Lactate level.
- Duration of mechanical ventilation.
- Pain, agitation and delirium.

Pain was assessed using the Visual Analog Scale or Behavioral Pain Scale (BPS) if the patient was deeply sedated. Agitation and delirium were assessed using the Richmond agitation-sedation scale and Confusion Assessment Method for ICU patients, respectively.

### Statistical analysis

SPSS software (version 23. IBM SPSS Statistics, Armonk, NY) was used for data analysis. Categorical data (gender, cause of ICU admission and source of sepsis) are herein presented as frequencies (%) and were analyzed using the Chi-square test. Continuous data are presented as the median (interquartile range: IQR). Baseline characteristics (age, BMI and APACHE II score) were analyzed using unpaired t tests. Hemodynamic data, levels of cytokines, and flow cytometry parameters were analyzed using mixed ANOVA between subject effect (treatment groups) and within subject effect (time) and post hoc Tukey’s test. A P value of 0.05 will be considered statistically significant.

### Sample size estimation

Power analysis was performed on the level of the mean CD42/CD14 24 h after study drug infusion. A previous study [5] reported that the mean CD42/CD14 in controls was 23%, with a standard deviation of 5%. On the basis of the assumption that a mean difference of 25% in CD42/CD14 was a clinically significant difference between groups, and for a power of 0.8 and an alpha error of 0.05, a minimum sample size of 12 patients was calculated for each group.

## Results

**Fig. 1:**
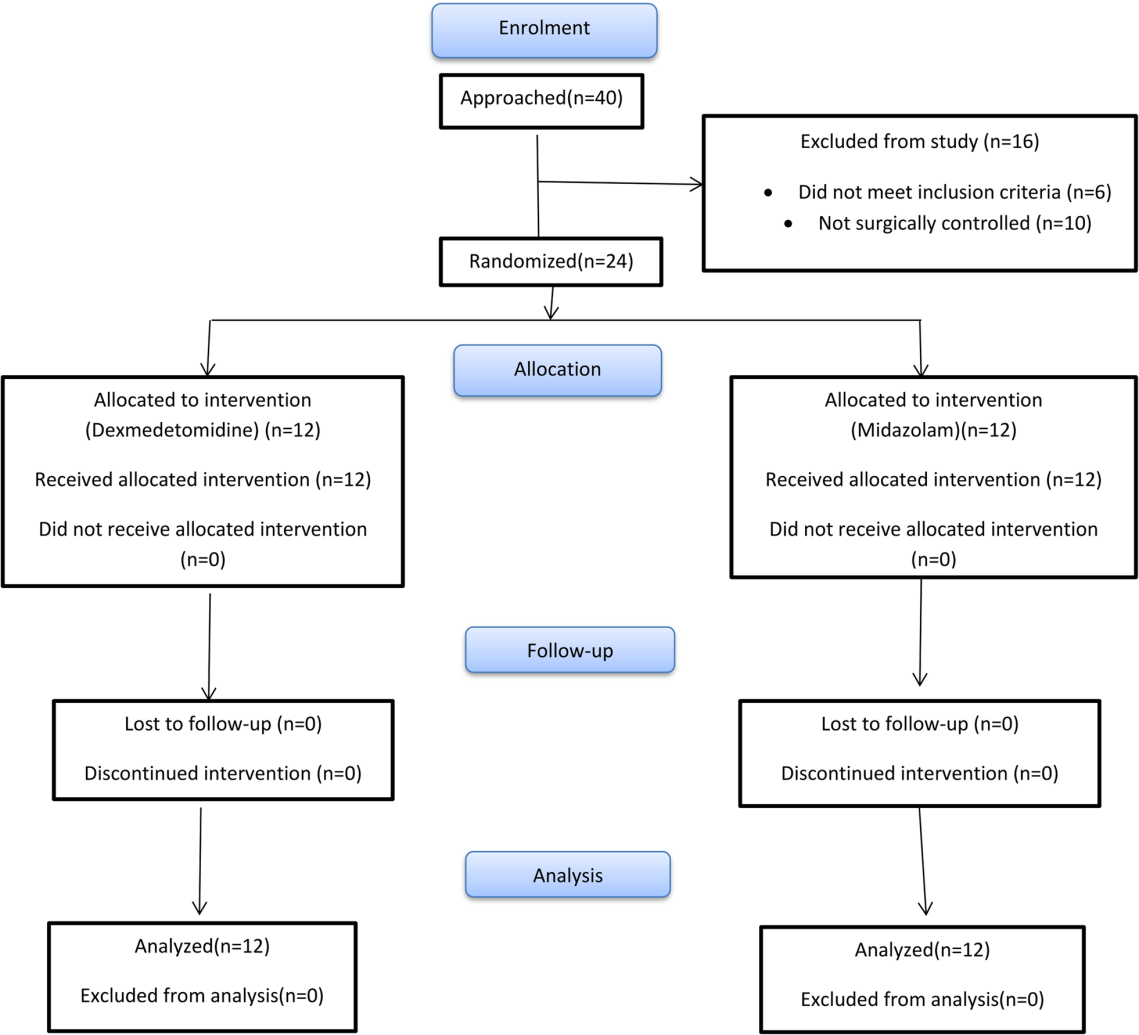
Patient flow chart

Patient enrollment started on June 20, 2019, and we approached 40 patients. Sixteen patients were excluded because they did not meet the inclusion criteria, and another ten patients were not surgically controlled. Twenty-four patients were randomized (Fig. 1).

**Fig. 2:**
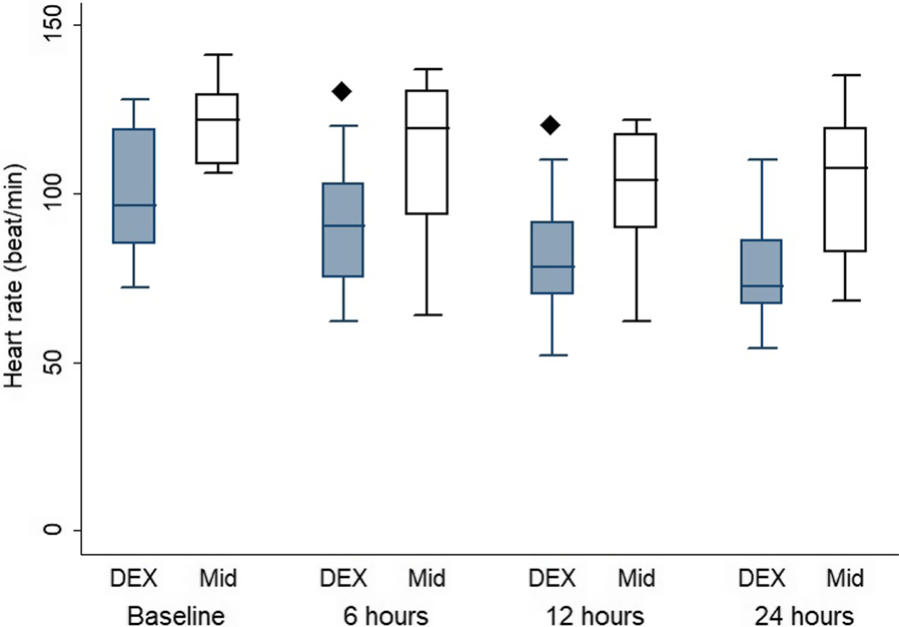
Heart rate comparison between the two groups. ♦ Denotes statistically significant

**Fig. 3:**
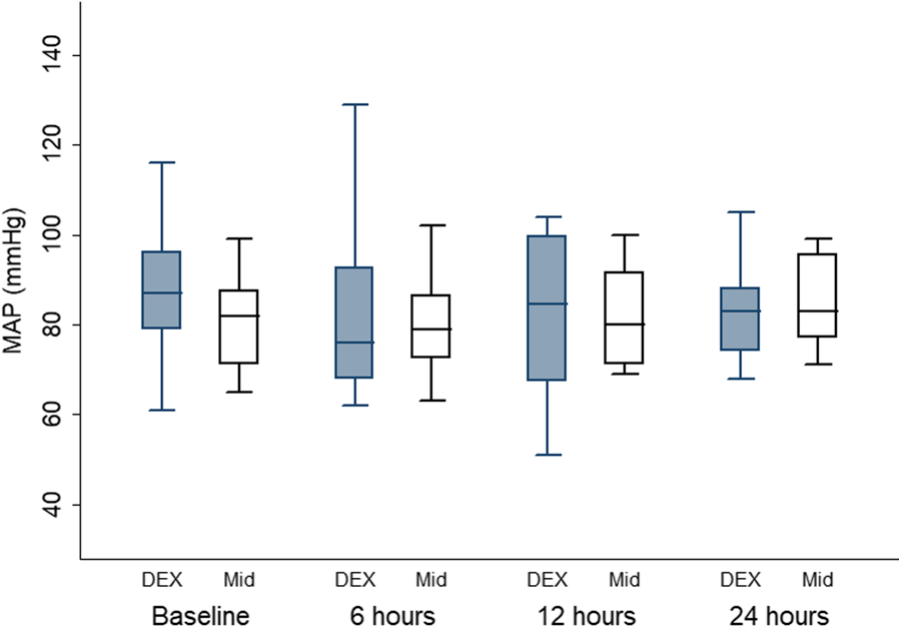
Mean arterial blood pressure comparison between the two groups

**Fig. 4:**
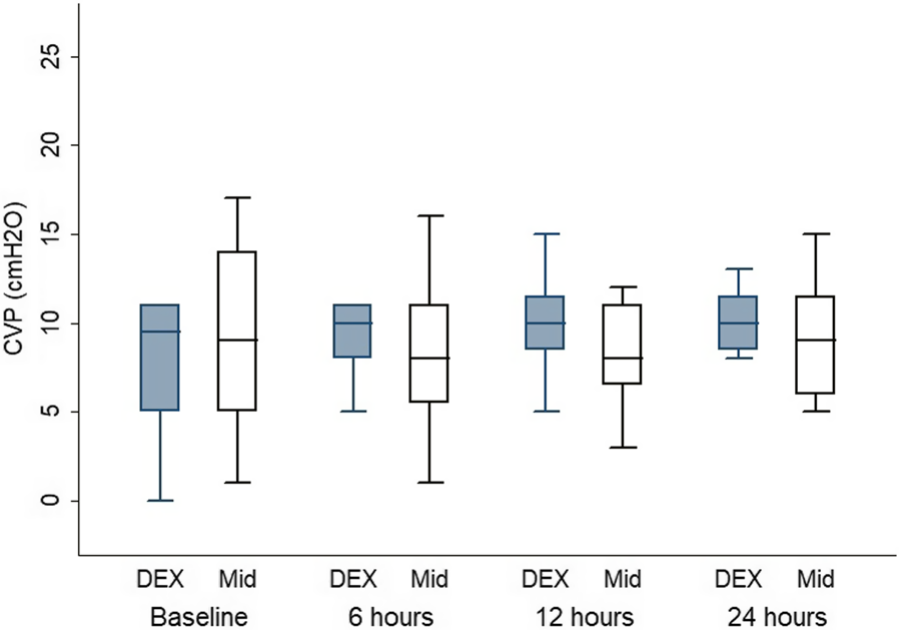
Central venous pressure comparison between the two groups

**Fig. 5:**
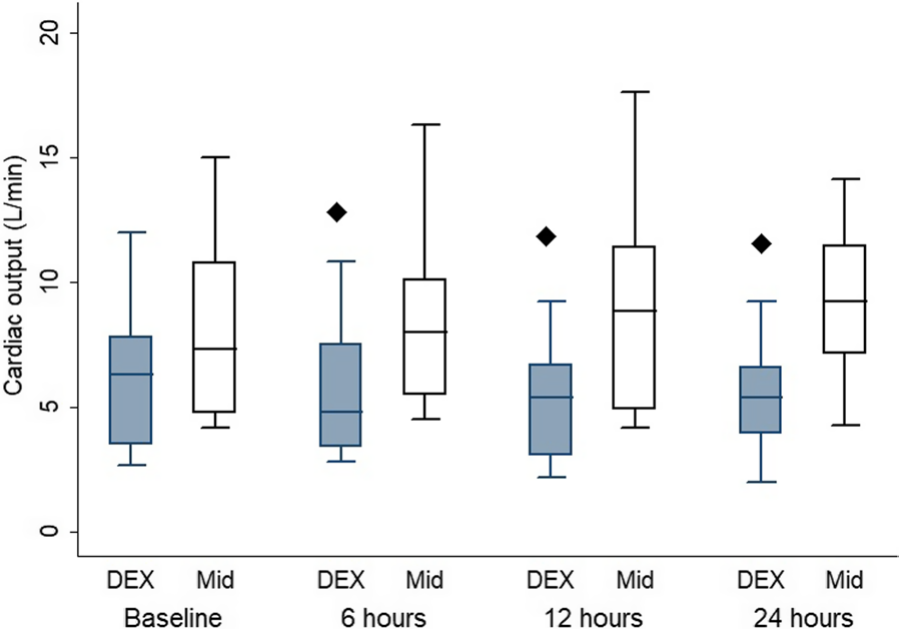
Cardiac output comparison between the two groups. ♦ Denotes statistically significant differences

The patients’ characteristics are presented in Table 1**,** with no statistically significant differences between the groups. The flow cytometry data of CD14/42b, CD42bMFI, CD14/HLA-DR and HLA-DR-MFI showed no significant difference between the two groups, as well as in the within-group comparison, at all time points compared with the baseline (Table 2). TLR had no significant difference between the two groups and in the within-group comparison at all time points compared with the baseline in the D group and a significant difference at 24 h in comparison to the baseline in the M group (p = 0.02) (Table 3). CD14/TLR and TLR-MFI showed no significant difference between the two groups and in the within-group comparison at all time points in comparison with the baseline in both groups (Table 3). IL6 and TREM1 had no significant difference between the two groups and in the within-group comparison at all time points in comparison with the baseline in both groups. **(**Table 3**).** HR revealed a significant difference between the two groups at 6 and 12 h (p = 0.03 and 0.009, respectively), but the within-group comparison showed a significant difference at 12 and 24 h in comparison with the baseline in the D group (p = 0.04 and 0.009, respectively) and a significant difference at 12 h in comparison to the baseline in the M group (p = 0.01) (Fig. 2). Regarding MAP (Fig. 3) and CVP (Fig. 4), there was no significant difference between the two groups and in the within-group comparison at all time points in comparison with the baseline in both groups. In CO (Fig. 5), over 24 h during the ICU stay, cardiac output decreased in the D group more than in the M group at 6, 12 and 24 h (p = 0.033, 0.021, 0.005, respectively), so there was a significant difference between the two groups. However, in the within-group comparison, there was no significant difference at all time points in comparison with the baseline in both groups. There was no difference between the groups in the SOFA score (Table 4). On the Behavioral Pain Scale, there was no significant difference between the two groups, but within-group comparisons showed significant differences at 6, 12 and 24 h in comparison with thebaseline in Group I (p = 0.002, < 0.001, < 0.009) and significant differences at 6, 12 and 24 h in comparison to the baseline in Group II (p =  < 0.001, < 0.001, < 0.001) (Table 4). Regarding the occurrence of bradycardia < 50, hypotension > 20%, the need to discontinue the drug, occurrence of AKI on admission, AKI during stay, dialysis and delirium, there was no significant difference between the groups (Table 5). Lactate showed no significant difference between the two groups, but the within-group comparison showed significant differences at 6, 12 and 24 h in comparison with the baseline in Group I (p = 0.02, 0.002, < 0.001) and significant differences at 12 and 24 h in comparison to the baseline in Group II (p =  < 0.001 and 0.002) **(**Table 6**).** There was no significant difference in the CRP level, duration of MV, duration of vasopressor use, ICU stay or hospital stay between the groups (Table 6).

**Table 1:** Patient characteristics

	Group D n = 12(%)	Group M n = 12(%)	P value
Gender			
Female	6 (50)	6 (50)	1
Male	6 (50)	6 (50)	
Age (yrs.)			
Mean ± SD	55.42 ± 10.2	50.75 ± 14	0.36
BMI			
Mean ± SD	31.75 ± 4.1	31.92 ± 4.6	0.92
APACHE II			
Mean ± SD	11.08 ± 4.7	11.33 ± 4.6	0.89
28 d-mortality			
Yes	3 (25)	4 (33.3)	1

Data are presented as the mean ± standard deviation and frequencies (%)

*BMI* body mass index

**Table 2:** CD14/42b, CD42b MFI1, CD14/HLA-DR and HLADR_MFI Data between the two groups

	Group D (n = 12) Median (IQR)	Group M (n = 12) Median (IQR)	P value	ANOVA P value
CD14/42b 0	14.2 (7–26.9)	6.9 (4.5–14.6)	0.33	0.87
CD14/42b 12	12.6 (4.1–30.9)	14.8 (6.2–19.2)	0.59	
CD14/42b 24	7 (4–19)	10.25 (6.1–13.2)	0.39	
CD42b MFI 0	9447.5 (5489.2–13,864)	6874.5 (3403–9161)	0.18	0.15
CD42b MFI 12	7727 (6391–14,584)	7706 (5074–10132)	0.27	
CD42b MFI 24	9708 (6179–11,072)	7270.5 (4099–8996.7)	0.22	
CD14/HLADR 0	8 (3.25–20.8)	10 (5.6–11.35)	0.97	0.64
CD14/HLADR 12	8.1 (4.6–15.9)	10.3 (3.9–17.4)	0.99	
CD14/HLADR 24	12.4 (1.7–19.02)	8 (4.5–13.3)	0.25	
HLA-DR-MFI 0	1844.5 (1342.5–2615)	1288.5 (844–2008.3)	0.12	0.21
HLA-DR-MFI 12	1316.5 (80.3–2288.3)	1172.5 (1019.3–1879.3)	0.33	
HLA-DR-MFI 24	1350 (762–2333.3)	1289 (1048.75–1398.5)	0.41	

Data are presented as the median (interquartile range)

*HLA* human leukocyte antigen, *MFI* mean fluorescent intensity

**Table 3:** TLR%, CD14/TLR4, TLR_MFI, IL6 and TREM1 data

	Group D (n = 12) Median (IQR)	Group M (n = 12) Median (IQR)	P value	ANOVA P value
TLR% 0	3.9 (2.85–13.75)	7.95 (3.2–12.5)	0.88	0.59
TLR% 12H	3.7 (1.7–14.42)	4.6 (2.87–8.12)	0.54	
TLR% 24H	3 (1.5–6.42)	3.6 (2.3–4.22)	0.26	
CD14/TLR4% 0	1.7 (1.2–7.22)	1.85 (0.4–3.6)	0.25	0.3
CD14/TLR4% 12H	1.8 (0.7–4.8)	1.7 (0.9–2.92)	0.45	
CD14/TLR4% 24H	0.95 (0.62–3.52)	1.45 (0.65–2.4)	0.5	
TLR-MFI 0	2998 (1053–6564.5)	1517 (951.3–4235.7)	0.2	0.67
TLR-MFI 12H	2163.5 (956.7–3637.25)	2068.5 (992–3043.25)	0.22	
TLR-MFI 24H	2011 (1433.75–5072.75)	2483 (1306.75–8458.7)	0.52	
IL6 0 (pg/mL)	351.4 (215.2–1012.3)	318.1 (131.2–1405.2)	0.84	0.74
IL6 12H (pg/mL)	97.6 (46.7–263.2)	185.8 (72.7–329.3)	0.39	
IL6 24H (pg/mL)	80.5 (37.1–135.6)	71 (51.8–163.7)	0.53	
TREM1 0 (pg/mL)	609.34 (248.9–1113.5)	653.6 (334–943.7)	0.97	0.18
TREM1 12H (pg/mL)	745.1 (318.3–875.5)	373.4 (220.75–782)	0.11	
TREM1 24H (pg/mL)	532.4 (240.6–1083.2)	442.3 (119.1–618.3)	0.12	

Data are presented as the median (interquartile range)

*TLR* Toll-like receptors, *MFI* Mean fluorescent intensity, *IL* Interleukin, *TREM1* Triggering receptor expressed on myeloid cells

**Table 4:** shows the SOFA score in 5 days during the ICU stay and the BPS between the two groups over 24 h

	Group D (n = 12) Median (IQR)	Group M (n = 12) Median (IQR)	P value	ANOVA P value
SOFA day 0	6 (5–7)	6 (5–7.75)	0.5	0.32
SOFA day 1	6 (5–7)	5.5 (3.5–7)	0.85	
SOFA day 2	6 (3.3–6.75)	6(3.3–7)	0.7	
SOFA day 3	4 (2.3–6)	6 (3.3–7.3)	0.16	
SOFA day 4	3 (3–6)	6 (3.3 -6.8)	0.13	
SOFA day 5	3 (2.25–6)	5 (3.25–6)	0.24	
BPS 0	5.5 (4–6)	6 (5–6)	0.06	0.04
BPS 6H	3.5 (3–4)	4 (3.3–4.8)	0.06	
BPS 12H	3 (3–4)	4 (3–4.8)	0.08	
BPS 24H	3 (3–4)	3.5 (3–5)	0.22	

Data are presented as the median (interquartile range)

*BPS* behavioral pain scale; *SOFA* sequential organ failure assessment score

**Table 5:** Patients showing bradycardia < 50, hypotension > 20%, drug discontinuation, AKI on admission, AKI during the hospital stay, delirium, and dialysis

	Group D (n = 12)	Group M (n = 12)	P value
Bradycardia < 50	1 (8.3%)	0(0.0%)	1
Hypotension > 20%	3(25.0%)	1(8.3%)	0.59
Drug discontinuation	0(0.0%)	0(0.0%)	–
AKI on admission	8(66.7%)	7(58.3%)	1
AKI during stay	1(8.3%)	0(0.0%)	1
Dialysis	0(0.0%)	2(16.7%)	0.47
Delirium	1 (8.3%)	0(0.0%)	1

Data are presented as frequencies and percentages

*AKI* acute kidney injury

**Table 6:** Lactate, CRP, duration of MV, duration of vasopressor use, ICU stay and hospital stay data between the two groups

	Group D (n = 12) Median (IQR)	Group M (n = 12) Median (IQR)	P value	ANOVA P value
Lactate 0 (mmole/l)	4.2 (3.12–5.5)	2.9 (2.4–3.9)	0.03	0.19
Lactate 6H (mmole/l)	2.3 (1.9–3.4)	2.3 (1.2–3.42)	0.8	
Lactate 12H (mmole/l)	1.9 (1.3–2.9)	1.5 (1.1–2.2)	0.32	
Lactate 24H (mmole/l)	1.4 (1.1–1.9)	1.15 (0.9–1.95)	0.97	
CRP 0 (mg/l)	267.5 (130.5–333.7)	147.5 (174.2–318.2)	0.72	0.71
CRP day 1 (mg/l)	290.5 (210.2–348.7)	245 (172–283.5)	0.31	
CRP Day 3 (mg/l)	188.5 (103–275.2)	203 (113.2–253.2)	0.68	
CRP Day 5 (mg/l)	173 (112.2–224.5)	181 (98.5–196)	0.85	
Duration of mechanical ventilation (days)	2.5 (1–3.75)	2 (1–6.75)	0.61	
Duration of vasopressors (days)	2 (1.25–4.5)	2 (1–3.75)	0.84	
ICU stay (days)	6.5 (5–7.75)	5.5 (5–8.5)	0.77	
Hospital stay (days)	8 (7–10)	7.5 (6–9)	0.45	

Data are presented as the median (interquartile range)

*CRP* C reactive protein

## Discussion

Sepsis is defined as life-threatening organ dysfunction due to a dysregulated host response to infection based on the definition of the Third International Consensus Definitions for Sepsis and Septic Shock (Sepsis 3.0). [6] The complex host immune response during sepsis involves the concomitant presence of both proinflammatory and anti-inflammatory responses but manifests disturbed homeostasis. It has been reported that the percentage of monocyte-platelet aggregation (CD42a/CD14 +) can reflect the level of inflammation and hemostasis, and the percentage of monocyte-activated cytokines (HLA-DR + /CD14 +) can reflect the state of immunosuppression. [7]

In this study, we found that there was no significant difference between dexmedetomidine and midazolam in either the proinflammatory pathway, as reflected by CD42a/CD14 + , or the anti-inflammatory pathway, as reflected by HLA-DR + /CD14 + levels, after 24 h of infusion in patients with septic shock. Contrary to our findings, Zhou et al. [5] examined the effect of dexmedetomidine on serum levels of CD42 + a/CD14 + and HLA-DR/CD14 + cells in patients undergoing multilevel spinal fusion and found that dexmedetomidine can inhibit the inflammatory response and enhance immunity by inhibiting the percentage of (CD42a+/CD14+), promoting the percentage of (HLA-DR+/CD14+) and reducing the production of proinflammatory cytokines, such as IL-6 and TNF-α. The difference between the study of Zhou et al. and our study may be related to the different types of patients.

Classically, the immune response in sepsis was traditionally envisioned as a biphasic sequela of events, namely, an initial hyperinflammatory reaction followed by a compensatory anti-inflammatory reaction that is responsible for immunoparalysis [8]. However, there is increasing evidence that immunoparalytic mechanisms exist from the onset of sepsis. The best biomarker to monitor immunoparalysis is HLA-DR because its expression specifically by monocytes is used to present pathogen antigens and activate T lymphocytes. Therefore, lower expression on the surface of monocytes (mHLA-DR) is related to a lower capacity of the immune system to respond to an infection. The unit of measurement of mHLA-DR can be the percentage of HLA-DR-positive monocytes CD14/HLA-DR (%) or the mean fluorescence intensity (MFI), the fluorescence unit relative to the monocyte population. Immunoparalysis is diagnosed when the percentage of HLA-DR-positive monocytes CD14/HLA-DR is < 30%. The cutoff value of HLA-DR MFI to diagnose immunoparalysis is not very clear in the literature. [9]. In our study, the median and IQR of CD14/HLA-DR at baseline were 8% (3%-20%) and 10% (5%-11%) in the dexmedetomidine and midazolam groups, respectively, which is diagnostic of immunoparalysis in our patients. Infusion of dexmedetomidine did not increase either CD14/HLA-DR or HLA-DR MFI. To the best of our knowledge, our study is the first study in humans to test the effect of dexmedetomidine on immunomodulation in septic patients. A previous animal study investigated the immunomodulatory effects of dexmedetomidine in a cecal ligation and puncture (CLP) model in rats. They found that dexmedetomidine partially induced immunomodulation with decreased HLA-DR and increased IL-6 production [10]. Several biomarkers, although nonspecific, could be used as surrogate markers of the proinflammatory and anti-inflammatory states of sepsis, such as CRP, TREM, TNF-α, IL-6, IL-10 and the TNF/IL-10 ratio. Our study revealed that there was no significant difference between the groups regarding TREM1, IL-6 and CRP. Several studies have investigated the role of dexmedetomidine in reducing the inflammatory response in surgical and septic patients and found that the use of dexmedetomidine decreased the production of inflammatory CRP, TNF-α and IL6 [11, 12]. A recent meta-analysis evaluated the effect of dexmedetomidine on the inflammatory response in the perioperative setting and found that dexmedetomidine decreased inflammatory markers such as IL6, TNF-α and C reactive protein (CRP) and increased other mediators such as CD4 + T cells, the ratios of CD4 + :CD8 + , natural killer cells and B cells [13].

The immunomodulatory mechanism of dexmedetomidine is not well known and may be multifactorial. Dexmedetomidine may modify cytokine production by macrophages and monocytes during the stress response [14, 15], and inhibit cellular apoptosis, which plays a main role in the pathogenesis of sepsis [16, 17]. Additionally, the stimulation of α2 adrenergic receptors by dexmedetomidine increases the phagocytic activity of macrophages, which may increase bacterial removal by the immune system [18, 19].

## Limitations

Our study had some limitations. First, the inflammatory response in patients was observed only for 24 h. Therefore, the effect of dexmedetomidine use for a longer duration in septic patients should be further explored. Second, we investigated the effect of dexmedetomidine on monocytes, and we did not explore its effect on other types of immune cells, such as neutrophils, dendritic cells, and natural killer cells. Third, we did not extend the infusion beyond 24 h. Extended infusion might have a positive effect on immune function. Last, the sample size was kept relatively small to address secondary outcomes and to adjust for confounders.

## Conclusion

Our results indicated that dexmedetomidine did not affect CD42a+/CD14+ and HLA-DR+/CD14+ expression in septic patients. Furthermore, cytokine production and inflammatory biomarkers did not change with dexmedetomidine infusion. Taken together, these findings did not support the hypothesis that dexmedetomidine has an immunomodulatory effect in patients with septic shock. Further studies are warranted to investigate extended infusion of dexmedetomidine beyond 24 h and to explore its effects on different types of immune cells.

## Data Availability

The data are available from the corresponding author on reasonable request.

## Abbreviation

BALF: Broncho-alveolar lavage fluid
BMI: Body mass index
CLP: Cecal perforation and ligation
CO: Cardiac output
CRP: C reactive protein
HLA: Human leukocyte antigen
IL: Interleukin
MAP: Mean arterial blood pressure
MFI: Mean fluorescent intensity
PMPs: Platelet-derived microparticles
RASS: Richmond Agitation and Sedation Scale
TLR: Toll-like receptors
TNF: Tumor necrosis factor
TREM1: Triggering receptor expressed on myeloid cells 1
